# Findings From the TechSAge Tele Tai Chi Clinical Trial

**DOI:** 10.1093/geroni/igaf122.242

**Published:** 2025-12-31

**Authors:** Tracy Mitzner, Elena Remillard, Kara Mumma

**Affiliations:** Person in Design, Atlanta, Georgia, United States; Georgia Institute of Technology, Atlanta, Georgia, United States; Georgia Institute of Technology, Atlanta, Georgia, United States

## Abstract

Tai chi is a mind-body exercise that involves a series of slow gentle movements, meditation, and controlled breathing. It has been shown to have a variety of evidence-based benefits, including improvements in balance, mobility, strength, flexibility, and relaxation, as well as reductions in falls, depression, and pain. While tai chi programs have grown in popularity and availability for older adults, many adults aging with mobility disabilities experience barriers participating in these exercise classes, including lack of transportation, appropriate exercise modifications, and building accessibility. The TechSAge Tele Tai Chi intervention was designed to address these barriers with 1) an evidence-based tai chi program (Tai Chi for Arthritis) that offers modifications appropriate for our target population (e.g., seated, movement adaptations) and 2) a translation to an interactive, online class via Zoom videoconferencing. We chose Tai Chi for Arthritis because it is a Title III-D (Highest Tier) Evidence-Based Health Promotion/Disease Prevention Program, endorsed by the Administration for Community Living. The intervention also includes moderated social time before and after the lesson as this population is at increased risk for social isolation. In this session, we will present outcomes from the 8-week clinical trial conducted with small-group cohorts of community-dwelling older adults aging with mobility disabilities (N = 60). Significant outcomes include increases in physical activity and social connectedness and decreases in loneliness. The findings of the clinical trial provide evidence that older adults with mobility disabilities can receive physical and social benefits from a group online tai chi program.

